# Cocaine

**Published:** 1897-04

**Authors:** 


### Cocaine.

Dr. Roswell Park, in his address at the University of Buffalo, on the "Introduction of Ether," had these good words to say for cocaine just before closing his remarks:

"Cocaine is now such a universally-recognized local anæsthetic that there is the best of reason for referring to it here—the more so because it affords another opportunity to do honor to a discoverer, who has rendered a most important service to not only our profession, but to the world in general. The principal active constituent of cocoa-leaves were discovered about 1860 by Niemann, and called by him cocaine. It is an alkaloid which combines with various acids in the formation of salts. It has the quality of benumbing raw and mucous surfaces, for which purpose it was applied first in 1862 by Schroff, and in 1868 by Moreno. In 1880, Van Aurap hinted that this property might some day be utilized. Karl Koller logically concluded, from what was known about it, that this anæsthetic property could be taken advantage of for work about the eye, and made a series of experiments upon the lower animals, by which he established its efficiency and made a brilliant discovery. He reported his experiments to the congress of German oculists, at Heidelberg, in 1884. News of this was transmitted with great rapidity, and within a few weeks the substance was used all over the world. Its use spread rapidly to other branches of surgery, and cocaine local anæsthesia became quickly an accomplished fact. More time was required to point out its disagreeable possibilities, its toxic properties, and the like; but it now has an assured and most important place among anæsthetic agents, and has been of the greatest use to, probably, 10 percent of the civilized world. To Koller is entirely due the credit of establishing its remarkable properties. Had he patented his discovery, he would have been vastly richer in pocket, though poorer in fame, than at present. He is now established in New York, where he enjoys a modest competency, but is, by no means, in receipt of the income which is properly his due from the world-at-large. To a man who has been the means of relieving so much pain as Karl Koller, no amount of pecuniary return is too great."

